# Role of gonadotropin-releasing hormone analogues in metastatic male breast cancer: results from a pooled analysis

**DOI:** 10.1186/s13045-015-0147-z

**Published:** 2015-05-17

**Authors:** Luigi Di Lauro, Laura Pizzuti, Maddalena Barba, Domenico Sergi, Isabella Sperduti, Marcella Mottolese, Carla Azzurra Amoreo, Franca Belli, Patrizia Vici, Valerie Speirs, Daniele Santini, Ruggero De Maria, Marcello Maugeri-Saccà

**Affiliations:** Division of Medical Oncology B, “Regina Elena” National Cancer Institute, Via Elio Chianesi 53, 00144 Rome, Italy; Scientific Direction, “Regina Elena” National Cancer Institute, Via Elio Chianesi 53, 00144 Rome, Italy; Biostatistics Unit, “Regina Elena” National Cancer Institute, Via Elio Chianesi 53, 00144 Rome, Italy; Department of Pathology, “Regina Elena” National Cancer Institute, Via Elio Chianesi 53, 00144 Rome, Italy; Division of Oncology, Spolverini Hospital, Ariccia, Italy; Leeds Institute of Cancer and Pathology, Wellcome Trust Brenner Building, University of Leeds, LS9 7TF Leeds, UK; Department of Medical Oncology, Campus Bio-Medico University of Rome, Rome, Italy

**Keywords:** Male breast cancer, Metastatic disease, Gonadotropin-releasing hormone analogue, Aromatase inhibitors, Cyproterone acetate

## Abstract

**Background:**

Male breast cancer is a rare malignancy. Despite the lack of prospectively generated data from trials in either the adjuvant or metastatic setting, patients are commonly treated with hormone therapies. Much controversy exists over the use of gonadotropin-releasing hormone analogues in metastatic male breast cancer patients. We conducted this study to provide more concrete ground on the use of gonadotropin-releasing hormone analogues in this setting.

**Methods:**

We herein present results from a pooled analysis including 60 metastatic male breast cancer patients treated with either an aromatase inhibitor or cyproterone acetate as a monotherapy (23 patients) or combined with a gonadotropin-releasing hormone analogue (37 patients).

**Results:**

Overall response rate was 43.5 % in patients treated with monotherapy and 51.3 % with combination therapy (*p* = 0.6). Survival outcomes favored combination therapy in terms of median progression-free survival (11.6 months versus 6 months; *p* = 0.05), 1-year progression-free survival rate (43.2 % versus 21.7 %; *p* = 0.05), median overall survival (29.7 months versus 22 months; *p* = 0.05), and 2-year survival rate (64.9 % versus 43.5 %; *p* = 0.05).

**Conclusions:**

In metastatic male breast cancer patients, the combined use of gonadotropin-releasing hormone analogues and aromatase inhibitors or antiandrogens seems to be associated with greater efficacy, particularly in terms of survival outcomes, compared with monotherapy. Collectively, these results encourage considering these agents in the metastatic setting.

## Background

Male breast cancer (MBC) is an uncommon malignancy accounting for less than 1 % of all breast cancer (BC) cases [1], albeit its incidence is rising [2]. The hormone-driven nature of the disease was postulated in the 1940s [2] and corroborated over the past decades by studies reporting on hormone receptor expression [3, 4]. Results from the National Cancer Institute’s Surveillance, Epidemiology, and End Results (SEER) database revealed that 92 % of MBC cases were estrogen receptor-positive [4]. Thus, antiestrogen therapy currently represents the mainstay of treatment for these patients, even though the use of tamoxifen [5], aromatase inhibitors (AIs) [6–8], and fulvestrant [9, 10] was investigated only retrospectively in small-sized cohorts. A therapeutic role for the androgen receptor (AR) was also envisioned [11–13] and corroborated by immunohistochemical analysis and gene-expression-profiling studies [3, 14]. Analysis of a large MBC cohort documented AR immunoreactivity in 64 % of cases [3], and over-expression of AR-related pathway components was reported [14].

Despite the wealth of hormonal treatments that have entered the therapeutic arena, owing to the rarity of this disease and lack of prospectively generated data, a number of unsolved questions afflict daily clinical practice. A heated argument surrounds the question of whether gonadotropin-releasing hormone analogues (GnRH analogues) are worth being administered in combination with other hormonal treatments acting on peripheral targets [7, 8, 13]. This controversy was fuelled by the advent of AIs [15]. In males, AIs lead to increased levels of follicle-stimulating hormone (FSH), luteinising hormone (LH), and testosterone (T) [16–20]. This phenomenon was observed in hypogonadal men and MBC patients [16–20]. For MBC patients, implications of increased T levels are twofold: i) the counteraction of the block imposed by AIs through an excess of substrate and ii) a direct stimulation of cancer cells equipped with the AR [21]. Briefly, the inhibition of the hypothalamic-pituitary feedback loop, with the correlated reduction of the substrate for aromatization, was the rationale for combining AIs with GnRH analogues. A second, though underestimated, association strategy relates to the use of GnRH analogues with antiandrogens [12, 13]. Our group reported on the antitumor activity of antiandrogens [11, 12], a finding we recently strengthened in a larger series where hints on the existence of an association between AR expression and clinical outcomes were also provided [13]. In this case, the use of antiandrogens with a GnRH analogue stemmed from the need to neutralize testicular and adrenal androgens, theorizing analogies in terms of androgen dependency between MBC and prostate cancer [13]. Indeed, our group already reported on the suppression of gonadotropins together with T suppression to castration levels in MBC patients who received cyproterone acetate (CPA) with buserelin [12]. These effects were also observed, although to a lower extent, with CPA monotherapy [11].

Therefore, there is a common theme underlying the use of GnRH analogue with antiandrogens and AIs, namely, achieving the deepest possible T suppression to directly or indirectly deprive cancer cells of a source of oncogenic stimuli, in the latter case by preventing the conversion of androstenedione to 17b-estradiol operated by the aromatase enzyme.

By evaluating metastatic MBC (mMBC) treated with an AI or CPA [8, 13], administered alone or combined with a GnRH analogue, we previously noted some differences favoring the association. Nevertheless, the relatively restricted number of patients analyzed hindered statistically significant comparisons. Prompted by this observation, we herein present results from a pooled analysis of these studies, with the inclusion of five additional patients, in order to gain more insights into the efficacy of GnRH analogue-containing hormonal therapy in mMBC patients.

## Results

Sixty men mostly treated in the first-line metastatic setting were included in the present analysis.

Patients’ characteristics are illustrated in Table 1. As shown in Table 1, the groups compared did not differ by any of the variables considered. Overall, 37 patients received GnRH analogue-containing therapy (22 patients with CPA and 15 patients with an AI), and 23 patients were treated with GnRH analogue-free therapy (14 patients with CPA and 9 patients with an AI).

**Table 1:** Association between clinical-pathological features and treatment received (N = 60)

Characteristic	With GnRH N (%)	Without GnRH N (%)	*p* value*
*Age*			
Median	64	63	*0.79*
Range	24–82	29–76	
*ECOG PS*			
Median	1	1	*0.88*
Range	0–2	0–2	
*Hormone receptor status*			
Positive	32 (86.5)	19 (82.6)	*0.99*
Negative	2 (5.5)	2 (8.7)	
Unknown	3 (8)	2 (8.7)	
*Adjuvant systemic therapy*			
Yes	19 (51)	10 (43.5)	*0.60*
No	18 (49)	13 (56.5)	
*Prior therapy for metastatic disease*			
Yes	9 (24)	7 (30.5)	*0.99*
No	28 (76)	16 (69.5)	
*Subsequent lines of CT*			
Median	1	1	*0.72*
Range	1–2	1–2	
*Subsequent lines of HT*			
Median	1	1	*0.85*
Range	1–3	1–3	
*Dominant disease site*			
Viscera	24 (64.9)	14 (60.8)	
Bone	9 (24.3)	8 (34.8)	*0.98*
Soft-tissue	4 (10.8)	1 (4.4)	
*Number of disease sites*			
1	11 (29.7)	10 (43.4)	
2	21 (56.7)	9 (39.1)	*0.99*
≥ 3	5 (13.6)	4 (17.5)	

ECOG PS: Eastern Cooperative Oncology Group Performance Status; CT: chemotherapy; HT: hormone therapy

*Fisher exact test

Stage at diagnosis was I, II, III, and IV in 11 (18.3 %), 20 (33.3 %), 21 (35 %), and 8 (13.4 %) patients, respectively. Stage I–II at diagnosis was 48 % in the monotherapy group and 52 % in the combination group. Overall, 29 patients received adjuvant systemic therapy, 10 in the monotherapy group (43.5 %) and 19 in the combination group (51 %). In the adjuvant setting, ten patients were treated with chemotherapy, ten patients with hormone therapy, and nine patients with both chemotherapy and hormone therapy. Adjuvant hormone therapy consisted of tamoxifen in all but one patient who received goserelin. Adjuvant chemotherapy consisted of cyclophosphamide, methotrexate, and 5-fluorouracil (CMF) in 11 patients; 5-fluorouracil, doxorubicin, and cyclophosphamide or 5-fluorouracil, epirubicin, and cyclophosphamide (FAC or FEC, respectively) in 5 patients; or epirubicin, cyclophosphamide, docetaxel (EC-D) in 3 patients. Sixteen patients received previous chemotherapy and/or hormone therapy for metastatic disease: 7 out of 23 (30.5 %) in the monotherapy group and 9 out of 37 (24 %) in the combination group. The characteristics of these treatments, along with clinical outcomes, were reported in detail elsewhere [8, 13]. Five patients treated in the first-line setting with an AI in monotherapy were retreated with an AI in association with a GnRH analogue after disease progression. These patients were included in the monotherapy group in the present analysis.

**Fig. 1:**
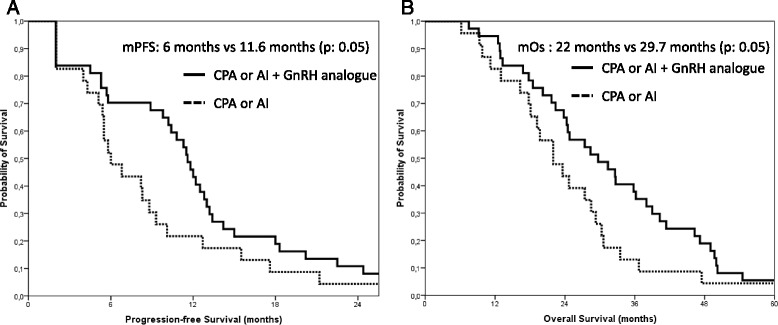
Kaplan–Meier curves of **a** PFS and **b** OS comparing monotherapy (CPA or AI) versus combination therapy containing a GnRH analogue

Overall response rate was 51.3 % (19/37 patients) in patients treated with combination versus 43.5 % with monotherapy (10/23 patients). The difference observed was not statistically relevant (*p* = 0.6). Median progression-free survival (mPFS) was 11.6 months (95 % CI = 10.2–13) in the group having received the GnRH analogue-containing combination and 6 months (95 % CI = 4–8) for patients treated with monotherapy (*p* = 0.05) (Fig. 1). One-year progression-free survival (PFS) rate also favored combination (43.2 % versus 21.7 %; *p* = 0.05). Median overall survival (mOS) was 29.7 months with combination (95 % CI = 20.4–39) and 22 months (95 % CI = 15.9–28.1) with monotherapy (*p* = 0.05) (Fig. 1). Two-year survival rate was 64.9 % in the combination group versus 43.5 % in the monotherapy group (*p* = 0.05).

## Discussion

Evidence on the therapeutic role of GnRH analogue as a partner for other hormonal agents in mMBC are scattered, gathered from case reports or small retrospective series, and overall inconsistent [7, 8, 13, 15]. More generally, therapeutic decision-making for mMBC is not built upon level I evidence, and without appearing nihilistic, we do not foresee brighter scenarios in the near future. Not surprisingly, then, there is no agreement on whether GnRH analogues should be considered an integral part of the therapeutic armamentarium, or rather, their use should be evaluated on a case-by-case basis [21, 22]. Anticipating that our results, which are retrospective in nature, are not intended to provide a definitive answer on that issue, to the best of our knowledge, this is the largest series presented so far describing the activity of GnRH analogue-containing therapy.

Before discussing our results, two introductive considerations need to be presented. As previously discussed elsewhere, we were unable to retrieve safety data for all patients included [8, 13]. However, when available, toxicities data were consistent with the expected frequency and severity. In addition, out-of-date imaging techniques and criteria for response evaluation were used in a fraction of patients.

First and foremost, in interpreting our results and analyzing the therapeutic potential of GnRH analogues, we would like to draw the reader’s attention to the line of reasoning that stimulated this study. We grouped patients treated with an AI or CPA as a monotherapy and compared them with patients that also received a GnRH analogue. As mentioned above, the logic behind this was the placement of T at the centerpiece of the endocrine network feeding MBC. Coherently, control of T levels is unsatisfactory in the case of exclusive use of CPA [23], or they even increase when AIs are administered in monotherapy [16–20]. Adding a GnRH analogue to peripherally acting antiestrogens and antiandrogens shares the same logic and produces the same output, that is, suppressing androgens [21, 23].

With the limitations of any retrospective and indirect comparison, we herein report a trend towards statistical significance favoring the use of GnRH analogue for all the survival endpoints considered. In order to put our results into context, it is worth considering that mPFS and mOS reported with combination therapy were fairly comparable with those previously observed with AIs, which were in the range of 4.4–13 and 33–39 months, respectively, independently on whether AIs were combined with a GnRH analogue or not [6–8]. These results are rooted, in our opinion, in the fact than more than half of the patients in this study received antiandrogen-based therapy mostly between the 1970s and 1980s [11–13]. With this therapeutic approach, mPFS was rather similar (8.9 months) to that observed with AIs, whereas mOS was shorter (24.3 months). Intuitively, this divergent pattern suggests that patients treated with antiandrogen-containing therapy were more likely to have received outdated post-progression treatments and possibly suboptimal supportive care, as already detailed [13]. In other words, the inclusion in the present analysis of patients treated at the dawning of antihormone therapy probably diluted the advantages potentially deriving from the use of GnRH analogues. Secondly and non-negligibly, five patients (~20 %), of whom four were previously presented in [8], included in the monotherapy group who received an AI as a first-line therapy were rechallenged with an AI plus a GnRH analogue following disease progression. Notably, four out five of them confirmed or improved the best overall response observed in the previous therapeutic line with AI monotherapy. We cannot therefore exclude a role for such a sequential approach in diluting treatment efficacy and ultimately flattening survival curves.

## Conclusions

We are aware that no firm conclusions can be drawn from this study, as the evidence provided does not meet criteria to settle the debate on the usefulness of GnRH analogues. On the other hand, however, clues emerging from this analysis encourage clinicians to consider GnRH analogues in the therapeutic continuum, irrespectively of the therapeutic “backbone” used. Finally, a provocative question arose that is whether, in pursuing the goal of sequential hormonal therapy for delaying chemotherapy, GnRH analogues shall deserve substantially increased consideration as singularly deliverable agents.

## Methods

In the present study, we evaluated a population of 60 mMBC patients who had received an AI or CPA mostly in the first-line setting, administered as a monotherapy or combined with a GnRH analogue. The majority of patients were clinically managed at the “Regina Elena” National Cancer Institute, Rome. Individual patient data were reviewed in order to retrieve information on demographic factors, molecular pathology, therapies, and treatment outcomes. Patients received the following treatments: letrozole 2.5 mg orally daily as a monotherapy or with leuprolide acetate or triptorelin acetate given intramuscularly at 3.75 mg every 28 days, CPA 100 mg twice a day as a monotherapy or combined with buserelin administered subcutaneously at 1500 μg daily in three doses during the first week and then reduced to 600 μg a day or Goserelin administered at 3.6 mg subcutaneously every 28 days, exemestane 25 mg once daily or anastrozole 1 mg once a day. Tumor response was evaluated according to the criteria outlined by the International Union Against Cancer [24], the World Health Organization [25], or the Response Evaluation Criteria In Solid Tumors (RECIST 1.1) coherently to the period when patients were treated. Progression-free survival (PFS) and overall survival (OS) were calculated from the date of therapy initiation to the date of disease progression or death from any cause, respectively. PFS and OS were analyzed according to the Kaplan–Meier method. Comparisons between groups were carried out with the Tarone–Ware test. Statistical analyses were performed using SPSS statistical software version 20 (SPSS inc., Chicago IL, USA). This study was approved by the Ethics Committee of “Regina Elena” National Cancer Institute of Rome and was carried out according to the Helsinki Declaration.

## Abbreviations

MBC: Male breast cancer
BC: Breast cancer
AIs: Aromatase inhibitors
AR: Androgen receptor
CMF: Cyclophosphamide, methotrexate, and 5-fluorouracil
GnRH analogues: Gonadotropin-releasing hormone analogues
EC-D: Epirubicin, cyclophosphamide, docetaxel
FAC: 5-Fluorouracil, doxorubicin, and cyclophosphamide
FEC: 5-Fluorouracil, epirubicin, and cyclophosphamide
FSH: Follicle-stimulating hormone
LH: Luteinising hormone
T: Testosterone
mMBC: Metastatic male breast cancer
CPA: Cyproterone acetate
PFS: Progression-free survival
OS: Overall survival.
